# Multivessel vs. culprit vessel-only percutaneous coronary intervention in ST-segment elevation myocardial infarction with and without cardiogenic shock

**DOI:** 10.3389/fcvm.2022.992456

**Published:** 2022-11-24

**Authors:** Jing Wu, Yonggang Wang, Chenguang Li, Honglei Ji, Wenyi Zhao, Qian Tong, Mingyou Zhang

**Affiliations:** ^1^Department of Translational Medicine, The First Hospital of Jilin University, Changchun, China; ^2^Department of Cardiovascular Diseases, The First Hospital of Jilin University, Changchun, China; ^3^Department of Cardiology, Zhongshan Hospital, Shanghai, China; ^4^Department of Nephrology, Linyi Traditional Chinese Medicine Hospital, Linyi, China

**Keywords:** STEMI, cardiogenic shock, culprit-only PCI, multivessel PCI, National Inpatient Database (NIS)

## Abstract

**Background:**

Early revascularization of the culprit vessel is the most effective treatment for reducing the risk of mortality from acute STEMI with and without cardiogenic shock. However, the most recent trends and impact of multivessel percutaneous coronary intervention (PCI) during the index hospitalization on in-hospital outcomes are unknown.

**Methods:**

The National Inpatient Sample was queried from October 2015 to 2019 for hospitalizations with STEMI. The impact of multivessel PCI on in-hospital outcomes of patients with and without cardiogenic shock was evaluated.

**Results:**

Of 624,605 STEMI hospitalizations treated with PCI, 12.5% were complicated by cardiogenic shock. Among hospitalizations without cardiogenic shock, 15.7% were treated by multivessel PCI, which declined from 20.8% in 2015 to 13.9% in 2019 (*P*_trend_ < 0.001). Multivessel and culprit-only PCI had similar rates of In-hospital mortality (2.4 vs. 2.3%, *p* = 0.027) and major adverse cardiac and cerebrovascular events (MACCE; 7.4 vs. 7.2%, *p* = 0.072). Among hospitalizations with cardiogenic shock, 22.1% were treated by multivessel PCI, which declined from 29.2% in 2015 to 19.4% in 2019 (*P*_trend_ < 0.001). Multivessel PCI was associated with higher rates of in-hospital mortality (30.9 vs. 28.4%, *p* < 0.001) and MACCE (39.9 vs. 36.5%, *p* < 0.001) than culprit-only PCI.

**Conclusion:**

The frequency of multivessel PCI for STEMI with and without cardiogenic shock is declining. Multivessel PCI is associated with worse in-hospital outcomes for STEMI with cardiogenic shock but not for STEMI without cardiogenic shock.

## Introduction

In patients presenting with ST-segment elevation myocardial infarction (STEMI) with or without cardiogenic shock, early revascularization—mainly percutaneous coronary intervention (PCI) on the culprit vessel—is the most effective therapeutic strategy to reduce both short- and long-term mortality (1–3). However, over half of patients with hemodynamically stable STEMI have at least 1 other obstructive lesion in non-culprit vessels (4); in STEMI with cardiogenic shock, up to 80% of patients present with multivessel coronary artery disease (5). Optimal strategies for the treatment of non-culprit lesions have been widely studied (6). Several randomized controlled trials including the COMPLETE trials comparing multivessel vs. culprit-only PCI have reported improved clinical outcomes including decreased cardiac mortality, myocardial reinfarction, and revascularization (7–12); however, the optimal time to treat non-culprit lesions is not known. Additionally, data supporting multivessel PCI have been derived from hemodynamically stable myocardial infarction (MI) patients as cardiogenic shock patients were excluded from these studies. The results of the CULPRIT-SHOCK trial enrolling patients with acute MI complicated by cardiogenic shock suggested that immediate treatment of non-culprit lesions during primary PCI was harmful (13). Real-world data regarding impact of multivessel PCI for STEMI with and without cardiogenic shock on in-hospital outcomes are limited and inconsistent (14–16). The most recent practice trends of multiple PCI are unknown, To address these issues, in this study we analyzed data for patients hospitalized for STEMI with and without cardiogenic shock using the latest United States (US) National Inpatient Sample (NIS) database.

## Materials and methods

### Data source

The data were obtained from the NIS database developed for the Healthcare Cost and Utilization Project (HCUP) (17). It is the largest inpatient care database in the US and includes over 7 million unweighted hospital stays annually with more than 100 clinical and non-clinical data elements. It is accessible at https://www.ahrq.gov. In accordance with NIS recommendations, proper weighting was applied using the individual weight variable provided by the HCUP to establish national estimate statistics. Comorbidities were identified using Elixhauser Comorbidity Software Refined for International Classification of Disease, 10th Revision, Clinical Modification (ICD-10-CM), which assigns data elements provided by HCUP. The use of the NIS database to describe outcomes and trends in cardiovascular disease in different patient populations has been previously validated (18, 19). As data from the NIS are publicly available and de-identified, the study was exempt from Institutional Review Board approval. The authors vouch for the accuracy and completeness of the data. the date of last database interrogation was on 10 July 2022.

### Study population and outcome measures

Starting from 1 October 2015, all hospitals in the US transitioned from ICD-9-CM (i.e., the 9th revision) to ICD-10-CM coding of diagnoses and procedures. Significant disruption of statistics has been reported, and it was suggested that analyses rely on a single coding system (20, 21). In the present study, we queried the NIS database from inception of the ICD-10-CM coding system to the latest available time (from October 2015 through 2019). STEMI hospitalizations were identified using the ICD-10-CM diagnosis codes I21.0x, I21.1x, I21.2x, and I21.3 (Supplementary Table 1), which have been previously validated (22, 23). We excluded records of patients who did not undergo PCI; with missing information on the number of treated vessels in procedure codes; with age at admission <18 years; and with missing data on in-hospital mortality (Supplementary Figure 1). The primary outcome was in-hospital all-cause mortality, and the secondary outcome was major adverse cardiac or cerebrovascular events (MACCE) including a composite of all-cause mortality, cardiac complications (hemopericardium and cardiac tamponade necessitating pericardiocentesis), and stroke. Hospital cost was obtained by merging the cost-to-charge ratio files with total charge.

### Statistical analyses

Continuous variables are expressed as mean ± SD or median [interquartile range (IQR)] as appropriate. Categorical variables are expressed as numbers and percentages. Weights for each discharge were used to calculate national estimates as recommended by the HCUP for NIS data. Multivariable logistic regression models were generated to evaluate the association between in-hospital mortality, presented as odds ratios (OR) with 95% confidence interval (CI), and variables included in the model (multivessel PCI, age, sex, race, expected payer, hospital bed size, location and teaching status, atrial fibrillation, smoking status, history of MI, prior PCI, prior coronary artery bypass graft [CABG], family history of coronary artery disease, chronic lung disease, obesity, peripheral artery disease, hypothyroidism, hypertension, and diabetes mellitus). Differences between categorical variables were evaluated with the chi-squared test, and differences between continuous variables were assessed with the Student’s *t*-test or Mann–Whitney *U* test as appropriate; the corresponding ORs and 95% CIs are presented as forest plots. The Breslow–Day test was used to analyze the interaction between subgroups. Considering the large sample size, a 2-sided *P*-value <0.01 was considered statistically significant. SAS 9.4 (SAS Institute, Cary, NC, USA) was used for all analyses.

## Results

### Temporal trends in multivessel percutaneous coronary intervention

**FIGURE 1:**
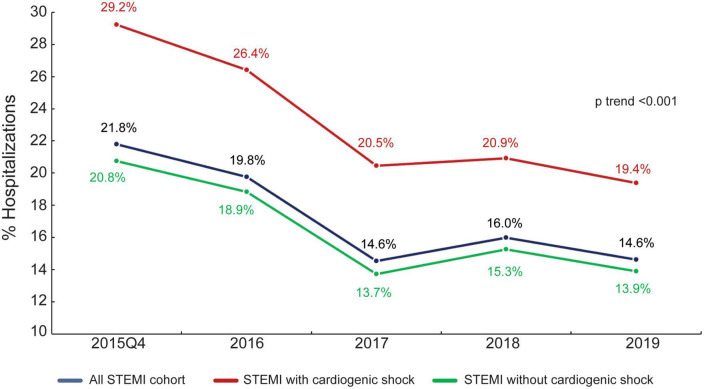
Trend of multivessel PCI performance during the study period. Percentage of overall STEMI hospitalizations, STEMI hospitalizations with cardiogenic shock, and STEMI hospitalizations without cardiogenic shock in which multivessel PCI was performed.

The flow chart of patient selection is shown in Supplementary Figure 1. We extracted 912,540 hospitalizations with a diagnosis of STEMI between October 2015 to October 2019 from the NIS database. After excluding age <18 years at admission (*n* = 440); patients with missing in-hospital mortality data (*n* = 3,300); hospitalizations did not undergo PCI (283,645); and hospitalizations with missing number of vessel treatments in procedure codes (*n* = 3,280), the final analysis included 624,605 STEMI hospitalizations, 546,305 (87.5%) without and 78,300 (12.5%) with cardiogenic shock. In the cohort without cardiogenic shock, there were 460,315 (84.3%) hospitalizations where the patient underwent culprit-only PCI and 85,990 (15.7%) where the patient underwent multivessel PCI. In the cohort with cardiogenic shock, there were 60,695 (77.9%) hospitalizations where the patient underwent culprit-only PCI and 17,335 (22.1%) where the patient underwent multivessel PCI. During the study period, the rate of multivessel PCI in overall STEMI hospitalizations declined from 21.8% in 2015 to 14.6% in 2019 (*P*_trend_ < 0.001); the rate of multivessel PCI for STEMI without cardiogenic shock declined from 20.8 to 13.9% (*P*_trend_ < 0.001); and the rate of multivessel PCI in hospitalizations with cardiogenic shock declined from 29.2 to 19.4% (*P*_trend_ < 0.001) (Figure 1).

**FIGURE 2:**
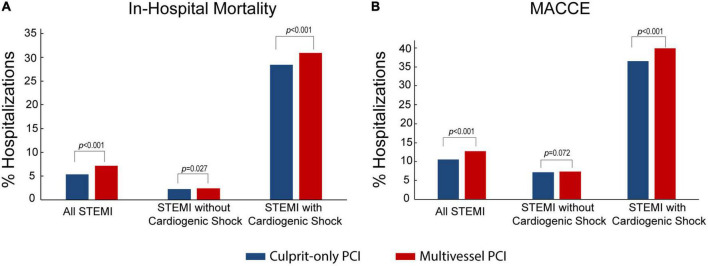
In-hospital mortality and MACCE in multivessel PCI vs. culprit-only PCI. **(A,B)** Shown are percentages of in-hospital mortality **(A)** and MACCE **(B)** comparing multivessel PCI vs. culprit-only PCI in the overall STEMI cohort, STEMI without cardiogenic shock cohort, and STEMI with cardiogenic shock cohort.

**FIGURE 3:**
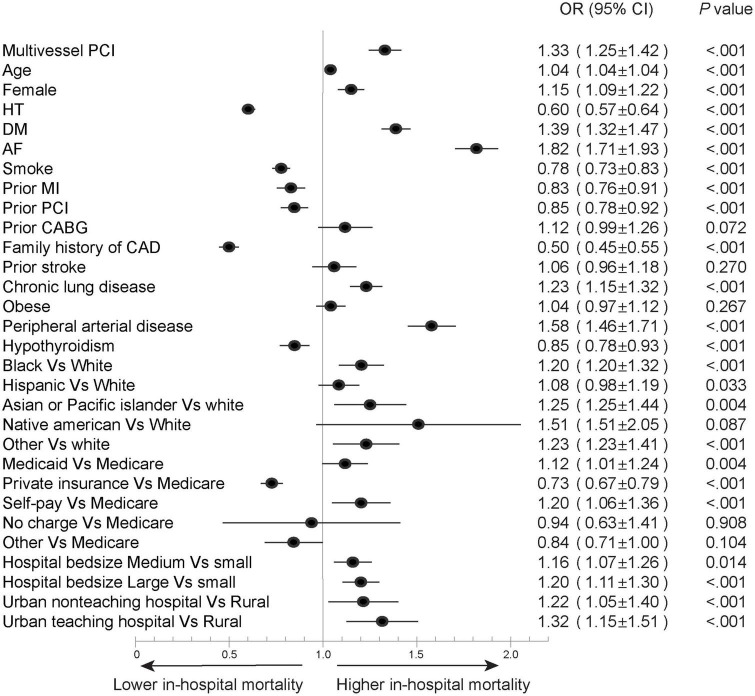
Forest plot of multivariable regression analysis to predict in-hospital mortality in overall STEMI cohort. AF, atrial fibrillation; CABG, coronary artery bypass graft; CAD, coronary artery disease; DM, diabetes mellitus; HT, hypertension; MI, myocardial infarction; PCI, percutaneous coronary intervention.

In the overall STEMI cohort, hospitalizations who underwent multivessel PCI were older (63.5 ± 12.2 vs. 62.5 ± 12.6) and mostly White (77.1 vs. 76.3%, *P* < 0.001); a higher proportion were males (73 vs. 70.9%, *P* < 0.001), and the patients had higher rates of hypertension (74.1 vs. 71.7%, *P* < 0.001), diabetes mellitus (34.5 vs. 30.9%, *P* < 0.001), and admission to a large hospital (56.1 vs. 54.5%, *P* < 0.001) compared to those who underwent culprit-only PCI. Multivessel PCI hospitalizations were associated higher rates of cardiogenic shock (16.8 vs. 11.7%, *P* < 0.001) and mechanical circulatory support (14.7 vs. 8.5%, *P* < 0.001), higher cost of care (35,980 ± 36,358 vs. 25,830 ± 22,514, *P* < 0.001), and longer hospital stay (median 3, IQR [2–5] vs. median = 3, IQR [2–4], *P* < 0.001) (Table 1); they also had higher in-hospital mortality compared to culprit-only PCI (7.2 vs. 5.4%, *P* < 0.001) and a higher rate of MACCE (12.8 vs. 10.6%, *P* < 0.001) (Figure 2). The increase in in-hospital mortality was observed in each calendar year during the study period (Supplementary Figures 2, 3). Logistic regression analysis in all STEMI cohorts showed that multivessel PCI was associated with increased risk for in-hospital mortality (OR = 1.33; 95% CI: 1.25–1.42, *p* < 0.001) (Figure 3). We categorized multivessel PCI into procedures involving 2 vessels and >2 vessels. The latter subgroup had higher rates of in-hospital mortality (9.6 vs. 6.8%, *P* < 0.001) and MACCE (15.8 vs. 12.3%, *P* < 0.001) compared to 2-vessel PCI (Supplementary Figure 4).

**TABLE 1:** Baseline characteristics in overall STEMI hospitalizations.

Variables	Culprit-only PCI *N* = 521,280	Multivessel PCI *N* = 103,325	*P*-value
Age	62.5 ± 12.6	63.5 ± 12.2	<0.001
Female	151,435 (29.1)	27,900 (27.0)	<0.001
Anterior STEMI	192,065 (36.8)	36,605 (35.4)	<0.001
Inferior STEMI	258,035 (49.5)	49,885 (48.3)	<0.001
Unspecified STEMI	79,105 (15.2)	19,990 (19.4)	<0.001
Race			<0.001
White	380,205 (76.3)	75,885 (77.1)	
Black	43,000 (8.6)	7,000 (7.1)	
Hispanic	41,185 (8.3)	8,180 (8.3)	
Asian/pacific islander	13,685 (2.8)	3,185 (3.2)	
Native American	2,635 (0.5)	565 (0.6)	
Other races	17,450 (3.5)	3,565 (3.6)	
Hypertension	373,945 (71.7)	76,560 (74.1)	<0.001
Diabetics	161,150 (30.9)	35,600 (34.5)	<0.001
History of smoke	140,915 (27.0)	27,970 (27.1)	0.805
Obesity	92,200 (17.7)	17,625 (17.1)	<0.001
Prior MI	62,685 (12.0)	12,370 (12.0)	0.630
Prior PCI	67,750 (13.0)	14,065 (13.6)	0.022
Prior CABG	18,770 (3.6)	4,330 (4.2)	<0.001
Prior stroke	24,305 (4.7)	5,090 (4.9)	<0.001
Peripheral arterial disease	37,245 (7.1)	8,475 (8.2)	<0.001
Chronic lung disease	73,755 (14.2)	13,870 (13.4)	<0.001
Hypothyroidism	43,240 (8.3)	8,480 (8.2)	0.675
Family history of CAD	81,195 (15.6)	16,265 (15.7)	0.549
Hospital size (number of beds)			<0.001
Small	79,735 (15.2)	14,995 (14.5)	
Medium	157,860 (30.3)	30,405 (29.4)	
Large	283,985 (54.5)	57,925 (56.1)	
Hospital location/teaching status			0.003
Rural hospital	31,220 (6.0)	6,405 (6.2)	
Urban non-teaching	119,000 (22.8)	23,870 (23.1)	
Urban teaching	310,060 (71.2)	73,050 (70.7)	
Payer			<0.001
Medicare	220,170 (42.3)	46,135 (44.7)	
Medicaid	56,500 (10.9)	10,590 (10.3)	
Private	185,740 (35.7)	35,585 (34.5)	
Self-pay	37,455 (7.2)	6,810 (6.6)	
No charge	3,140 (0.6)	565 (0.6)	
Other	17,295 (3.3)	3,445 (3.3)	
Systemic thrombolysis	10,905 (2.1)	2,500 (2.4)	<0.001
Thrombectomy	76,475 (14.7)	14,060 (13.6)	<0.001
MCS	44,370 (8.5)	15,145 (14.7)	<0.001
Cardiogenic shock	60,965 (11.7)	17,335 (16.8)	<0.001
Cost of care, U.S. $	25,830 ± 22,514	35,980 ± 36,358	<0.001
Length of hospital stay	2 (2, 4)	3 (2, 5)	<0.001

Values are mean ± SD, n (%), or median (interquartile range). CABG, coronary artery bypass grafting; CAD, coronary artery disease; MCS, mechanical circulatory support; MI, myocardial infarction; PCI, percutaneous coronary intervention; STEMI, ST segment elevation myocardial infarction.

**FIGURE 4:**
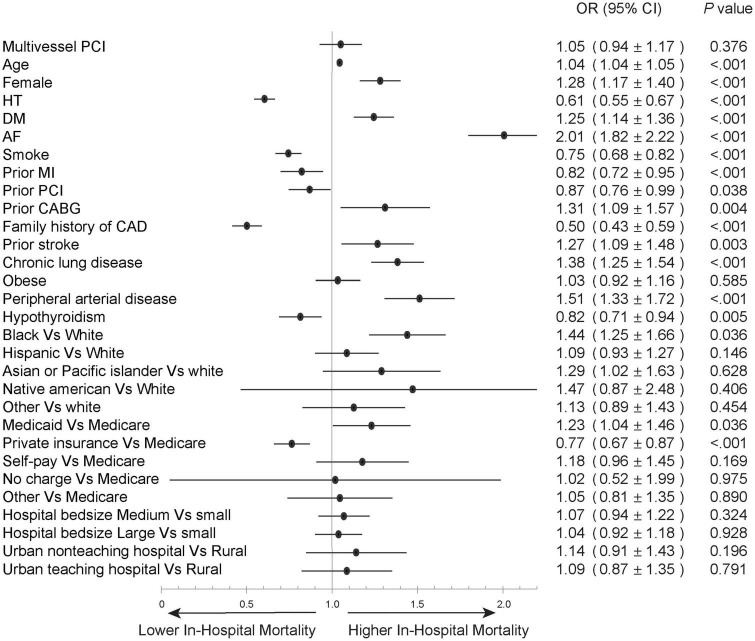
Forest plot of multivariable regression analysis to predict in-hospital mortality in the STEMI without cardiogenic shock cohort. Abbreviations as in Figure 3.

We next compared the impact of multivessel PCI with and without cardiogenic shock. Among hospitalizations without cardiogenic shock, hospitalizations underwent multivessel PCI are were older (63.0 ± 12.2 vs. 62.1 ± 12.5, *P* < 0.001) and mostly male (73.5 vs. 71.5%, *P* < 0.001) and White (77.6 vs. 76.5%, *P* < 0.001) (Table 2), with higher rates of hypertension (75.0 vs. 72.3%, *P* < 0.001), diabetes mellitus (33.3 vs. 30.5%, *P* < 0.001), and admission to a large hospital (55.3 vs. 54.3%, *P* < 0.001). Multivessel PCI hospitalizations were associated with a higher cost of care (30,691 ± 25,804 vs. 22,990 ± 15,863, *P* < 0.001) and longer hospital stay (median = 3, IQR [2–4] vs. median 2, IQR [2–3], *P* < 0.001), but had in-hospital mortality (2.4 vs. 2.3%, *P* = 0.027) and MACCE rate (7.4 vs. 7.2% *P* = 0.072) similar to culprit-only PCI hospitalizations (Figure 2). The rate of in-hospital mortality was comparable between the two groups in each calendar year during the study period (Supplementary Figures 2, 3). Logistic regression analysis showed that in the STEMI without cardiogenic shock cohort, multivessel PCI was not associated with an increased risk of in-hospital mortality (RR = 1.05; 95% CI:0.94–1.17) (Figure 4). When we stratified the procedure into 2-vessel and >2-vessel PCI (Supplementary Figure 4), the 2-vessel procedure had in-hospital mortality (2.3 vs. 2.3%) and MACCE rate (7.2 vs. 7.1%) similar to culprit-only PCI; however, PCI involving >2 vessels was associated with worse in-hospital outcomes (in-hospital mortality, 3.3% and MACCE, 8.8%). Additionally, although other subgroups showed comparable in-hospital mortality risk, >2-vessel PCI was associated with an increased risk of in-hospital death (OR = 1.45, 95% CI: 1.15–1.82) (Supplementary Figure 5).

**TABLE 2:** Baseline characteristics in STEMI hospitalizations without cardiogenic shock.

Variables	Culprit-only PCI *N* = 460,315	Multivessel PCI *N* = 85,990	*P*-value
Age	62.1 ± 12.5	63.0 ± 12.2	<0.001
Female	131,160 (28.5)	22,745 (26.5)	<0.001
Anterior STEMI	165,860 (36.0)	28,700 (33.4)	<0.001
Inferior STEMI	231,020 (50.2)	43,215 (50.3)	0.712
Unspecified STEMI	70,030 (15.2)	16,450 (19.1)	<0.001
Race			<0.001
White	336,805 (76.5)	63,570 (77.6)	
Black	38,285 (8.7)	5,905 (7.2)	
Hispanic	36,165 (8.2)	6,635 (8.1)	
Asian/pacific islander	11,630 (2.6)	2,445 (3.0)	
Native American	2,235 (0.5)	465 (0.6)	
Other races	15,030 (3.4)	2,920 (3.6)	
Hypertension	332,565 (72.3)	64,500 (75.0)	<0.001
Diabetics	140,250 (30.5)	28,620 (33.3)	<0.001
History of smoke	126,375 (27.5)	24,000 (27.9)	0.006
Obesity	82,300 (17.9)	14,770 (17.2)	<0.001
Prior MI	56,110 (12.2)	10,465 (12.2)	0.873
Prior PCI	60,940 (13.2)	11,970 (13.9)	<0.001
Prior CABG	16,610 (3.6)	3,755 (4.4)	<0.001
Prior stroke	21,265 (4.6)	4,170 (4.9)	0.003
Peripheral arterial disease	30,580 (6.6)	6,290 (7.3)	<0.001
Chronic lung disease	62,705 (13.6)	10,930 (12.7)	<0.001
Hypothyroidism	37,690 (8.2)	6,905 (8.0)	0.121
Family history of CAD	75,310 (16.4)	14,645 (17.3)	<0.001
Hospital size (number of beds)			<0.001
Small	71,040 (15.4)	12,705 (14.8)	
Medium	139,570 (30.3)	25,705 (29.9)	
Large	249,705 (54.3)	47,580 (55.3)	
Hospital location/teaching status			0.003
Rural hospital	28,060 (6.1)	5,530 (6.4)	
Urban non-teaching	105,650 (23.0)	20,220 (23.5)	
Urban teaching	326,605 (71.0)	60,240 (70.1)	
Payer			<0.001
Medicare	188,520 (41.0)	36,860 (43.0)	
Medicaid	50,275 (10.9)	8,665 (10.1)	
Private	168,430 (36.7)	31,000 (36.1)	
Self-pay	33,745 (7.3)	5,850 (6.8)	
No charge	2,850 (0.6)	510 (0.6)	
Other	15,665 (3.4)	2,940 (3.4)	
Systemic thrombolysis	9,280 (2.0)	2,075 (2.4)	<0.001
Thrombectomy	64,265 (14.0)	10,910 (12.7)	<0.001
MCS	13,455 (2.9)	4,230 (4.9)	<0.001
Cost of care, U.S. $	22,990 ± 15,863	30,691 ± 25,804	<0.001
Length of hospital stay	2 (2, 3)	3 (2, 4)	<0.001

Values are mean ± SD, n (%), or median (interquartile range). Abbreviations as in Table 1.

**FIGURE 5:**
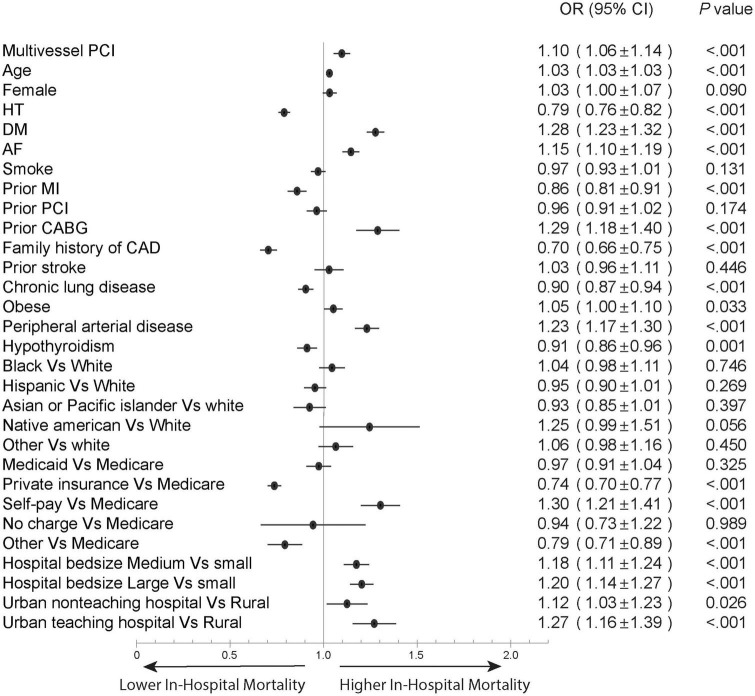
Forest plot of multivariable regression analysis to predict in-hospital mortality in the STEMI with cardiogenic shock cohort. Abbreviations as in Figure 3.

The mean ± SD age of patients hospitalized with cardiogenic shock was similar between those who underwent multivessel vs. culprit-only PCI (66.0 ± 11.9 vs. 65.9 ± 12.3, *P* = 0.871); however, the former cohort had more men (70.2 vs. 66.7%, *P* < 0.001) (Table 3) and higher rates of hypertension (69.6 vs. 67.9%, *P* < 0.001), diabetes mellitus (40.3 vs. 34.3%, *P* < 0.001), and admission to a large hospital (59.7 vs. 56.2%, *P* < 0.001). Multivessel PCI hospitalizations were associated with a higher cost of care (62,225 ± 136,938 vs. 47,273 ± 97,822, *P* < 0.001), longer hospital stay (median = 5, IQR [2–10] vs. median = 5, IQR [2–9], *P* < 0.001), and higher in-hospital mortality (30.9 vs. 28.4%, *P* < 0.001) and MACCE rate (39.9 vs. 36.5%, *P* < 0.001) than culprit-only PCI (Figure 2). The in-hospital mortality in each calendar year during the study period is shown in the Supplementary Figure 2. Logistic regression revealed that for STEMI with cardiogenic shock, multivessel PCI was associated with increased risk of in-hospital mortality (OR = 1.10; 95% CI: 1.06–1.14) (Figure 5). In the subgroup analysis, the rate of in-hospital mortality for 2-vessel and >2-vessel procedures were 30.7 and 31.6%, respectively, and the rate of MACCE was 39.8 and 40.3%, respectively, with similar results observed across all subgroups (Supplementary Figure 6).

**TABLE 3:** Baseline characteristics in STEMI hospitalizations with cardiogenic shock.

Variables	Culprit only PCI *N* = 60,965	Multiple vessels PCI *N* = 17,335	*P*-value
Age	65.9 ± 12.3	66.0 ± 11.9	0.871
Female	20,275 (33.3)	5,155 (29.8)	<0.001
Anterior STEMI	26,205 (43.0)	7,905 (45.6)	<0.001
Inferior STEMI	27,015 (44.3)	6,670 (38.5)	<0.001
Unspecified STEMI	9,075 (14.9)	3,540 (20.4)	<0.001
Race			<0.001
white	43,400 (74.8)	12,315 (74.9)	
black	4,715 (8.1)	1,095 (6.7)	
Hispanic	5,020 (8.7)	1,545 (9.4)	
Asian/pacific islander	2,055 (3.5)	740 (4.5)	
Native American	400 (0.7)	100 (0.6)	
Other races	2,420 (4.2)	645 (3.9)	
Hypertension	4,1380 (67.9)	12,060 (69.6)	<0.001
Diabetics	20,900 (34.3)	6,980 (40.3)	<0.001
History of smoke	14,540 (23.9)	3,970 (22.9)	0.010
Obesity	9,900 (16.2)	2,855 (16.5)	0.468
Prior MI	6,575 (10.8)	1,905 (11.0)	0.445
Prior PCI	6,810 (11.2)	2,095 (12.1)	<0.001
Prior CABG	2,160 (3.5)	575 (3.3)	0.153
Prior stroke	3,040 (5.0)	920 (5.3)	0.089
Peripheral arterial disease	6,665 (10.9)	2,185 (12.6)	<0.001
Chronic lung disease	11,050 (18.1)	2,940 (17.0)	<0.001
hypothyroidism	5,550 (9.1)	1,575 (9.1)	0.942
Family history of CAD	5,885 (9.7)	1,620 (9.4)	0.224
Hospital size (number of beds)			<0.001
Small	8,394 (13.8)	2,290 (13.2)	
Medium	18,290 (30.0)	4,700 (27.1)	
large	34,280 (56.2)	10,345 (59.7)	
Hospital location/teaching status			0.036
Rural hospital	3,160 (5.2)	875 (5.1)	
Urban non-teaching	13,350 (21.9)	3,650 (21.1)	
Urban teaching	44,455 (72.9)	12,810 (73.9)	
Payer			<0.001
Medicare	31,650 (52.0)	9,275 (53.6)	
Medicaid	6,225 (10.2)	1,925 (11.1)	
Private	17,310 (28.5)	4,585 (26.5)	
Self-pay	3,710 (6.1)	960 (5.6)	
No charge	290 (0.5)	55 (0.3)	
Other	1,630 (2.7)	505 (2.9)	
Systemic thrombolysis	1,625 (2.7)	425 (2.5)	0.120
Thrombectomy	12,210 (20.0)	3,150 (18.2)	<0.001
MCS	30,915 (50.7)	10,915 (63.0)	<0.001
Cost of care, U.S. $	47,273 ± 97,822	62,225 ± 136,938	<0.001
Length of hospital stay	5 (2, 9)	5 (2, 10)	<0.001

Values are mean ± SD, n (%), or median (interquartile range). Abbreviations as in Table 1.

## Discussion

There were five main findings from this large-sample analysis of patients with STEMI with or without cardiogenic shock in the US. (1) The rate of multivessel PCI in the index hospitalization decreased during the study period, corresponding to the declining rates of STEMI with and without cardiogenic shock. (2) In the overall STEMI cohort, in-hospital mortality and rate of MACCE for multivessel PCI were significantly higher than the rate of culprit-only PCI. (3) In STEMI hospitalizations without cardiogenic shock, multivessel PCI was not associated with an elevated risk of in-hospital mortality and MACCE rate. (4) In STEMI hospitalizations with cardiogenic shock, multivessel PCI was associated with a significantly increased risk of in-hospital mortality and MACCE rate. (5) The elevated risk of multivessel PCI in the overall STEMI cohort was driven by the higher portion of cardiogenic shock hospitalizations in which patients underwent multivessel PCI, and higher risk associated with multivessel PCI in cardiogenic shock hospitalizations.

Multivessel disease is common in STEMI hospitalizations, and even more prevalent in the setting of cardiogenic shock (5). The presence of multivessel disease is associated with worse clinical outcomes compared with single-vessel disease (24). The optimal strategy for treatment of the non-culprit vessel is unclear, as reflected in the discrepancies in treatment guidelines. The current evidence indicates diverse effects of multivessel PCI on clinical outcomes depending on the presence of cardiogenic shock. Except for the CULPRIT-SHOCK trial, randomized clinical trials have excluded patients with cardiogenic shock and have reported favorable outcomes of multivessel PCI, with earlier trials showing that the benefit was mainly attributable to a reduction in repeated revascularizations (7–10, 25–29). The COMPLETE trial (11) showed that the benefit extended beyond repeated revascularizations, also reducing the rates of cardiac death and MI (12). However, the optimal timing of non-culprit vessel revascularization has not been adequately investigated. An analysis of 1,964 patients from 5 clinical trials that included multivessel PCI during the index hospitalization demonstrated a significant reduction in cardiovascular mortality in addition to repeated revascularizations (12). The present analysis of NIS data confirms the safety of non-culprit PCI during the index hospitalization for STEMI without cardiogenic shock.

During the study period, multivessel PCI was performed during the index hospitalization in only 15.7% of STEMI hospitalizations without cardiogenic shock; thus, most patients with multivessel disease admitted with STEMI did not have their non-culprit vessel treated before discharge. Although, the clinical benefit of non-culprit PCI has been established (11), several questions remain unanswered, like what is the optimal timing of non-culprit PCI (30), our data provide support for the treatment of non-culprit vessel coronary disease during the index hospitalization, considering the possible long-term benefit for complete revascularization (31). Thus, for STEMI without cardiogenic shock, multivessel PCI during the index hospitalization appears safe and should be considered, at least in selected hemodynamically stable myocardial infarction patients. However, it is worth noting that in this analysis, 85.7% of multivessel procedures were performed on two vessels. The 2-vessel procedure is safe and does not incur excessive risks of in-hospital mortality and MACCE compared with culprit-only PCI. Hospitalizations involving a >2-vessel procedure is still associated with a significant increase in in-hospital mortality and MACCE. These results indicate that there is a limit to how many vessels can be safely treated. In cases involving >2 vessels, it is important to consider whether staged PCI to treat the extra vessel(s) is more beneficial or whether CABG is a better option because of the complexity of the coronary artery disease.

The results of the CULPRIT-SHOCK trial showed the detrimental effect of immediate multivessel PCI on cardiogenic shock complicated by MI at 30 days (13). In line with this finding, our analysis showed that in contrast to STEMI hospitalizations without cardiogenic shock, multivessel PCI was associated with increased risk of in-hospital mortality and MACCE in STEMI hospitalizations with cardiogenic shock. An explanation for the differential impact of multivessel PCI in hospitalizations with vs. without cardiogenic shock is that the long procedure time may cause more stress and expose patients to more hemodynamic instability; additionally, injection of a large amount of contrast agent may further impair the function of an underperfused kidney in the setting of cardiogenic shock. To our knowledge, this analysis represents the largest-sample study of the impact of multivessel PCI on STEMI with cardiogenic shock. Our results provide real-world evidence of the harmful effects of immediate multivessel PCI as reported in the CULPRIT-SHOCK trial. The declining trend of multivessel PCI performance in the setting of cardiogenic shock during the study period may reflect the influence of the CULPRIT-SHOCK trial on clinical practice. It has been suggested that immediate multivessel PCI is associated with a higher short-term but lower long-term risk of death than culprit lesion-only PCI; however, this is not supported by the 1-year outcome from the CULPRIT-SHOCK trial that showed no reduction in the multivessel PCI group with a longer follow-up (between 30 days and 1 year) (32). This along with our findings suggest that immediate multivessel PCI should be avoided in STEMI with cardiogenic shock. In the CULPRIT-SHOCK trial, staged PCI of non-culprit lesions within 30 days was only performed on 17.4% patients. Whether performing more stage PCIs can improve outcomes and if so, the optimal time to treat the non-culprit lesion remain to be determined.

### Study limitations

The present analysis had certain limitations. Large in-patient cohorts such as the NIS are subject to coding and documentation errors. The administrative database lacked clinical details for individual hospitalization including angiographic and procedural details, biochemistry data, echocardiography, and medications as well as long-term follow-up data; moreover, the retrospective observational study design made the analysis liable to selection bias. However, the NIS database has been widely validated internally and externally in studies with adequate sampling (33). Our analyses were robust and included subgroup analyses; moreover, they included the most current and largest sample of patients with STEMI with cardiogenic shock and provides insight into the practice patterns and impact of multivessel PCI in the real world, confirming the findings of the CULPRIT-SHOCK trial.

## Conclusion

In this national analysis of STEMI hospitalizations with and without cardiogenic shock, we found a significant decrease in the performance of multivessel PCI for STEMI both with and without cardiogenic shock in the US from 2015 to 2019. In STEMI admissions without cardiogenic shock, PCI of no more than 1 non-culprit vessel can be safely performed during the index hospitalization. However, in STEMI with cardiogenic shock, multivessel PCI during the index hospitalization was associated with increased risks of in-hospital mortality and MACCE. Further study is needed to determine whether patients with STEMI with cardiogenic shock benefit from staged multivessel PCI, and the optimal procedure time thereof.

## Data availability statement

The raw data supporting the conclusions of this article will be made available by the authors, without undue reservation.

## Ethics statement

Ethical review and approval was not required for the study on human participants in accordance with the local legislation and institutional requirements. Written informed consent for participation was not required for this study in accordance with the national legislation and the institutional requirements.

## Author contributions

MZ: full access to all the data in the study and responsible for the integrity of the data and the accuracy of the data analysis. MZ and QT: concept and design. JW, YW, MZ, and QT: acquisition, analysis, or interpretation of data. JW and YW: drafting of the manuscript and funding acquisition. JW and MZ: statistical analysis. HJ and WZ: administrative, technical, or material support. All authors: critical revision of the manuscript for important intellectual content.

## Abbreviations

CABG: coronary artery bypass graft
HCUP: Healthcare Cost and Utilization Project
ICD-9/10-CM: International Classification of Disease, 9th/10th Revision, Clinical Modification
MACCE: major adverse cardiac and cerebrovascular event
MI: myocardial infarction
NIS: National Inpatient Sample
PCI: percutaneous coronary intervention
STEMI: ST-segment elevation myocardial infarction.
