# Supplementary material for: Multivessel vs. culprit vessel-only percutaneous coronary intervention in ST-segment elevation myocardial infarction with and without cardiogenic shock
Source: Front Cardiovasc Med. 2022 Nov 24;9:992456. doi: 10.3389/fcvm.2022.992456 (PMC9731335; doi:10.3389/fcvm.2022.992456)
Supplement: Supplementary file 1 [file Data_Sheet_1.docx]

**Supplementary Appendix**

**Multivessel versus culprit vessel-only PCI in STEMI with and without cardiogenic shock**

**Table of contents of the supplementary appendix:**

**Supplementary Table**

**supplementary Figures**

**Supplementary table. ICD10 diagnosis and procedure codes**

| Anterior STEMI |
| --- |
| I21.01 I21.02 I21.09 |
| Inferior STEMI |
| I21.11 I21.19 |
| Unspecified STEMI |
| I21.21 I21.29 I21.3 |
| Smoking status |
| Z87.891 F17.200 F17.201 F17.203 F17.208 F17.209 F17.210 F17.211 F17.213 F17.218 F17.219 F17.220 F17.221 F17.223 F17.228 F17.229 F17.290 F17.291 F17.293 F17.298 F17.299 Z72.0 |
| Previous MI |
| I25.2 |
| Previous PCI |
| Z98.61 Z95.5 |
| Previous CABG |
| Z95.1 |
| Family history of CAD |
| Z82.49 Z82.41 |
| Previous Stroke |
| Z86.73 |
| Right heart angiography |
| B2040ZZ B2041ZZ B204YZZ B2140ZZ B2141ZZ B214YZZ |
| Left heart angiography |
| B2050ZZ B2051ZZ B205YZZ B2150ZZ B2151ZZ B215YZZ |
| Right and Left heart angiography |
| B2060ZZ B2061ZZ B206YZZ B2160ZZ B2161ZZ B216YZZ B2000ZZ B2001ZZ B200YZZ B2010ZZ B2011ZZ B201YZZ B2100ZZ B2101ZZ B210YZZ B2110ZZ B2111ZZ  B211YZZ 4A023N7 4A023N8 |
| PCI procedure |
| 0210344 02103D4 0211344 02113D4 02123D4 0270346 027034Z 0270356 027035Z 0270366 027036Z 0270376 027037Z 02703D6 02703DZ 02703E6 02703EZ 02703F6 02703FZ 02703G6 02703GZ 02703T6 02703TZ 02703Z6 02703ZZ 0271346 027134Z 0271356 027135Z 0271366 027136Z 0271376 027137Z 02713D6 02713DZ 02713E6 02713EZ 02713F6 02713FZ 02713G6 02713GZ 02713T6 02713TZ 02713Z6 02713ZZ 0272346 027234Z 0272356 027235Z 0272366 027236Z 0272376 027237Z 02723D6 02723DZ 02723EZ 02723F6 02723FZ 02723G6 02723GZ 02723TZ 02723Z6 02723ZZ 0273346 027334Z 0273356 027335Z 0273366 027336Z 0273376 027337Z 02733D6 02733DZ 02733EZ 02733FZ 02733GZ 02733Z6 02733ZZ 02C03Z6 02C03ZZ 02C13Z6 02C13ZZ 02C23Z6 02C23ZZ 02C33Z6 02C33ZZ 02H03DZ 02H13DZ 02H23DZ 02Q03ZZ 02Q13ZZ 02Q23ZZ 02U03JZ 02U13JZ X2C0361 X2C1361 X2C2361 X2C3361 |
| Cardiogenic shock |
| R57.0 |
| Hemopericardium |
| I31.2 |
| Pericardiocentesis |
| 0W9D30Z 0W9D3ZX 0W9D3ZZ 0W9D40Z 0W9D4ZX 0W9D4ZZ 0W9D0ZX 0W9D0ZZ |
| Cardiac tamponade |
| I31.4 |
| Cerebral infarction |
| G43.601 G43.609 G43.611 G43.619 I63.00 I63.011 I63.012 I63.013 I63.019 I63.02 I63.031 I63.032 I63.033 I63.039 I63.09 I63.10 I63.111 I63.112 I63.113 I63.119 I63.12 I63.131 I63.132 I63.133 I63.139 I63.19 I63.20 I63.211 I63.212 I63.213 I63.219 I63.22 I63.231 I63.232 I63.233 I63.239 I63.29 I63.30 I63.311 I63.312 I63.313 I63.319 I63.321 I63.322 I63.323 I63.329 I63.331 I63.332 I63.333 I63.339 I63.341 I63.342 I63.343 I63.349 I63.39 I63.40 I63.411 I63.412 I63.413 I63.419 I63.421 I63.422 I63.423 I63.429 I63.431 I63.432 I63.433 I63.439 I63.441 I63.442 I63.443 I63.449 I63.49 I63.50 I63.511 I63.512 I63.513 I63.519 I63.521 I63.522 I63.523 I63.529 I63.531 I63.532 I63.533 I63.539 I63.541 I63.542 I63.543 I63.549 I63.59 I63.6 I63.8 I63.81 I63.89 I63.9 |
| Hemorrhagic stroke |
| I60.00 I60.01 I60.02 I60.10 I60.11 I60.12 I60.2 I60.20 I60.21 I60.22 I60.30 I60.31 I60.32 I60.4 I60.50 I60.51 I60.52 I60.6 I60.7 I60.8 I60.9 I61.0 I61.1 I61.2 I61.3 I61.4 I61.5 I61.6 I61.8 I61.9 I62.00 I62.01 I62.02 I62.03 I62.1 I62.9 |
| Gastrointestinal bleeding |
| K92.0 K92.1 K92.2 |

CABG: Coronary artery bypass grafting; CAD: coronary artery disease; MI: myocardial infarction; PCI: Percutaneous coronary intervention; STEMI: ST segment elevation myocardial infarction

**Supplementary Figure 1.** Study flow diagram of hospitalization selection.


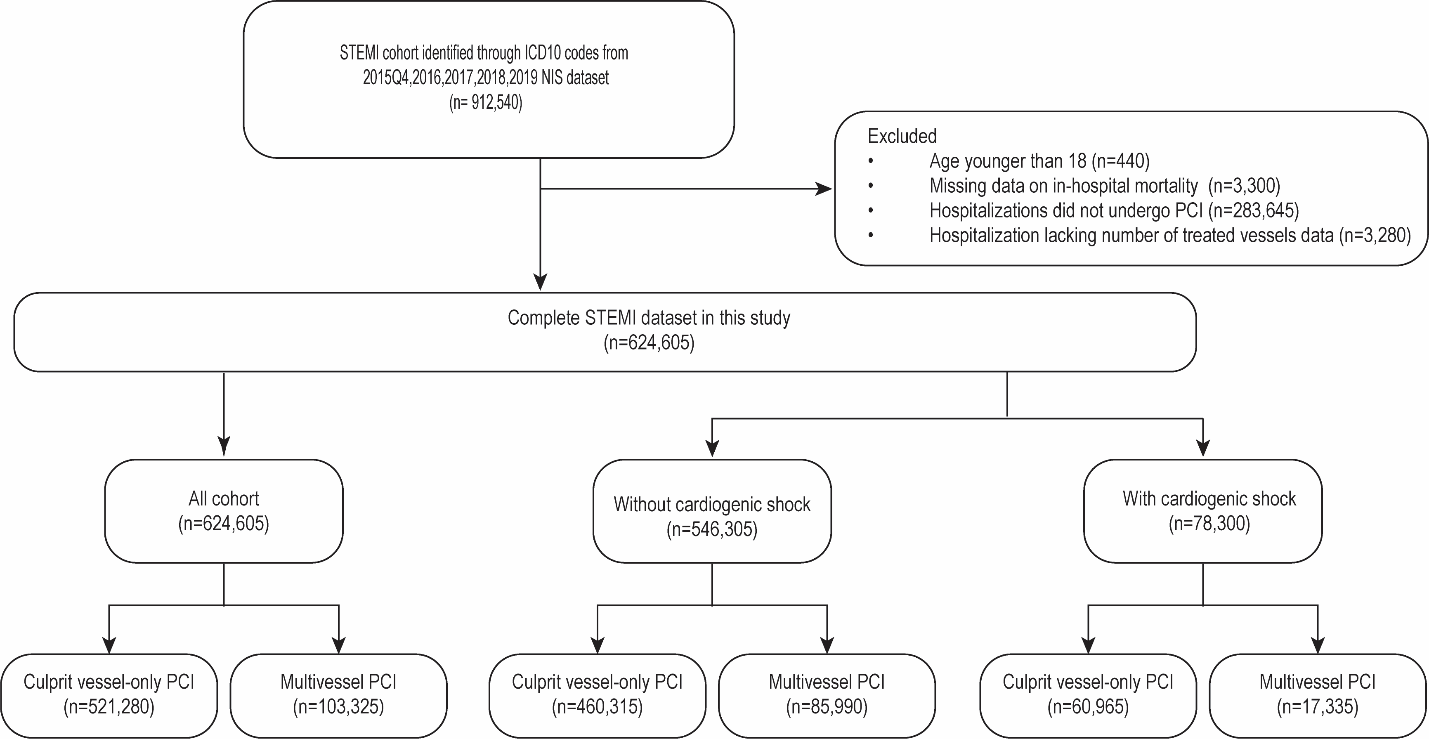


ICD, International Classification of Disease; NIS, National Inpatient Sample; PCI, percutaneous coronary intervention; STEMI, ST segment elevation myocardial infarction.

**Supplementary Figure 2. Overall and annual rates of In-hospital Mortality.**

Shown are percentages of In-hospital Mortality for overall STEMI cohort (Panel A), for the STEMI without cardiogenic shock (Panel B), and STEMI with cardiogenic shock (Panel C).

STEMI denotes ST segment elevation myocardial infarction.

**Supplementary Figure 3. Overall and annual rates of MACCE.**

Shown are percentages of MACCE for overall STEMI cohort (Panel A), for the STEMI without cardiogenic shock (Panel B), and STEMI with cardiogenic shock (Panel C). MACCE denotes major adverse cardiac and cerebrovascular events.

**Supplementary Figure 4. In-hospital mortality and MACCE for 3 groups analysis**

Shown are percentages of In-hospital mortality (Panel A) and MACCE (Panel B) for overall STEMI cohort, STEMI with and without cardiogenic shock categorized by 1 vessel, 2 vessels and more than 2 vessels groups.

**Supplementary Figure 5. Subgroup analysis for STEMI hospitalization without cardiogenic shock.**

**Supplementary Figure 6. Subgroup analysis for STEMI hospitalization with cardiogenic shock.**
